# Total neoadjuvant therapy followed by a watch-and-wait strategy for patients with rectal cancer (TOWARd): protocol for single-arm phase II/III confirmatory trial (JCOG2010)

**DOI:** 10.1093/bjsopen/zrad110

**Published:** 2023-11-01

**Authors:** Tadayoshi Hashimoto, Shunsuke Tsukamoto, Keiko Murofushi, Yoshinori Ito, Hidekazu Hirano, Yuichiro Tsukada, Keita Sasaki, Junki Mizusawa, Haruhiko Fukuda, Atsuo Takashima, Yukihide Kanemitsu

**Affiliations:** Japan Clinical Oncology Group Data Centre/Operations Office, National Cancer Centre Hospital, Tokyo, Japan; Translational Research Support Section, National Cancer Centre Hospital East, Kashiwa, Japan; Department of Gastroenterology and Gastrointestinal Oncology, National Cancer Centre Hospital East, Kashiwa, Japan; Department of Colorectal Surgery, National Cancer Centre Hospital, Tokyo, Japan; Division of Radiation Oncology, Tokyo Metropolitan Cancer and Infectious Disease Centre Komagome Hospital, Tokyo, Japan; Department of Radiation Oncology, Showa University School of Medicine, Tokyo, Japan; Department of Gastrointestinal Medical Oncology, National Cancer Centre Hospital, Tokyo, Japan; Department of Colorectal Surgery, National Cancer Centre Hospital East, Kashiwa, Japan; Japan Clinical Oncology Group Data Centre/Operations Office, National Cancer Centre Hospital, Tokyo, Japan; Japan Clinical Oncology Group Data Centre/Operations Office, National Cancer Centre Hospital, Tokyo, Japan; Japan Clinical Oncology Group Data Centre/Operations Office, National Cancer Centre Hospital, Tokyo, Japan; Department of Gastrointestinal Medical Oncology, National Cancer Centre Hospital, Tokyo, Japan; Department of Colorectal Surgery, National Cancer Centre Hospital, Tokyo, Japan

## Abstract

**Background:**

Radical surgery is the standard treatment for rectal cancer, but can impact quality of life. Recently, the concept of total neoadjuvant therapy with a watch-and-wait strategy has been proposed in which patients with a cCR after total neoadjuvant therapy do not proceed to surgery. However, most investigations of a watch-and-wait strategy have reported cases where cCR was achieved coincidentally via total neoadjuvant therapy. The aim is to assess whether total neoadjuvant therapy is effective in early-stage rectal cancer in patients that achieve cCR and are offered a watch-and-wait strategy.

**Methods:**

JCOG2010 (TOWARd) is a multi-institutional, single-arm phase II/III confirmatory investigation of the safety and efficacy of total neoadjuvant therapy followed by a watch-and-wait strategy for rectal cancer. Key eligibility criteria include cT2–3 N0 M0 rectal adenocarcinoma, tumour diameter less than or equal to 5 cm, age 18–75 years, performance status 0–1, and no history of pelvic irradiation or rectal surgery. Total neoadjuvant therapy involves neoadjuvant chemoradiotherapy (capecitabine and radiotherapy: 45 Gy/25 fractions to the whole pelvis plus boost of 5.4 Gy/3 fractions to the primary tumour) followed by consolidation chemotherapy (four cycles of capecitabine/oxaliplatin). Patients will be re-staged every 8 weeks after total neoadjuvant therapy, and those who achieve cCR will undergo a watch-and-wait strategy, those with near complete response will undergo a watch-and-wait strategy or local resection, and those with an incomplete response will undergo radical surgery. The primary endpoint is the cCR rate in phase II and 5-year overall survival in phase III. Secondary endpoints include postoperative anal, urinary, and sexual function. A total of 105 patients (phase II, 40 patients; phase III, 65 patients) will be enrolled over 3.5 years.

**Conclusion:**

This trial will determine whether total neoadjuvant therapy and a watch-and-wait strategy is an effective alternative to radical surgery for early-stage rectal cancer in patients with cT2–3 N0 M0 and tumour size less than or equal to 5 cm.

**Registration number:**

jRCTs031220288 (https://jrct.niph.go.jp/en-latest-detail/jRCTs031220288).

## Teaser

The concept of total neoadjuvant therapy with a watch-and-wait strategy has been put forward in recent years, in which surgery is not required in patients who have a cCR after total neoadjuvant therapy. However, patients who are suitable for a watch-and-wait strategy and the cCR rate achieved by total neoadjuvant therapy remain to be determined. Therefore, JCOG2010 was initiated, a multi-institutional, single-arm phase II/III trial that is designed to confirm that total neoadjuvant therapy followed by a watch-and-wait strategy is non-inferior to the standard therapy of surgical resection for rectal cancer.

## Introduction

Rectal cancer (RC) is the eighth leading cause of cancer-related mortality worldwide^1^. The standard treatment for cT2–3 N0 M0 RC in Japan is surgical resection followed by postoperative chemotherapy. However, some patients with RC who undergo surgery experience a considerable decline in postoperative quality of life (QoL), including frequent defaecation, need for a temporary/permanent colostomy as a result of rectal resection, and urinary or sexual dysfunction as a consequence of autonomic nerve damage during the procedure^2,3^. The perioperative treatment strategy for stage II/III RC varies among different guidelines. The European Society for Medical Oncology (ESMO) guidelines recommend upfront total mesorecta excision for T3a/b Nany^4^, whereas the American Society of Clinical Oncology (ASCO) guidelines recommend preoperative chemotherapy with radiation for patients with elevated risk profiles of local regional recurrence in the pelvis^5^. Furthermore, the concept of total neoadjuvant therapy (TNT) followed by a watch-and-wait strategy (W&W) has been put forward in recent years, in which surgery is not required in patients who have a cCR after TNT^6–9^. However, the cCR rate has varied considerably in different trials due to variability in the target population and in the response evaluation criteria used. Therefore, patients who are suitable for W&W and the cCR rate achieved by TNT remain to be determined.

TNT, which includes neoadjuvant chemoradiation and preoperative chemotherapy, is presently being advanced in the USA and Europe with the objective of improving the local anti-tumour effect by integrating neoadjuvant chemoradiotherapy (nCRT) and the distant anti-tumour effect by systemic chemotherapy. A previous investigation assessed multiple courses of a modified FOLFOX (fluorouracil/leucovorin plus oxaliplatin) regimen for consolidation chemotherapy and found that the pCR rate in resected specimens increased with the duration of treatment^10^. Therefore, in the upcoming JCOG2010 study, TNT rather than nCRT alone will be used to increase the pCR rate. In the CAO/ARO/AIO-12 trial, which compared the efficacy of induction *versus* consolidation chemotherapy in patients with locally advanced RC, the pCR rate was higher in the consolidation chemotherapy group than in the induction chemotherapy group (27 *versus* 19 per cent respectively) and the incidence of grade 3–4 adverse events was higher in the induction chemotherapy group than in the consolidation chemotherapy group (37 *versus* 27 per cent respectively)^11,12^. Therefore, the JCOG2010 study will investigate TNT with nCRT followed by consolidation chemotherapy.

A phase III trial conducted in Germany demonstrated that oral capecitabine was non-inferior to 5-fluorouracil/leucovorin for locally advanced RC^13^. Capecitabine is the chemotherapy most used in combination with radiotherapy worldwide, including in Japan^14^. Therefore, JCOG2010 will use oral capecitabine alone as the chemotherapy regimen in combination with radiotherapy. In terms of radiotherapy, the National Comprehensive Cancer Network guidelines suggest that the pelvic cavity should be irradiated at a dose of 45 Gy/25 fractions with primary tumour boost irradiation at 5.4 Gy/3 fractions as the treatment approach in nCRT^15^, which will be used in this study. With regard to the consolidation chemotherapy regimen, the CAO/ARO/AIO-12 trial, which administered six cycles of FOLFOX after nCRT, reported that 22 per cent of patients experienced grade 3 or higher adverse events during consolidation chemotherapy. Although there are no reports comparing FOLFOX with CAPOX (capecitabine/oxaliplatin) as a preoperative treatment, both are commonly used in clinical practice and are effective as adjuvant chemotherapy for colon cancer^16^. In view of its convenience, CAPOX has been chosen as the protocol treatment in this trial. FOLFOX is administered as six cycles at 2-week intervals over 12 weeks and CAPOX as four cycles at 3-week intervals over 12 weeks. Given the comparable efficacy of these regimens as adjuvant chemotherapy, four cycles of CAPOX will be used as consolidation chemotherapy in the JCOG2010 study.

The aim of this multi-institutional, single-arm phase II/III trial is to examine the safety and efficacy of TNT followed by W&W in patients with early-stage RC and high potential for non-operative management.

## Methods

### Overall study design

JCOG2010 is a multi-institutional, single-arm phase II/III trial that is designed to confirm that TNT followed by W&W is non-inferior to the standard therapy of surgical resection with lymph node dissection for cT2–3 N0 M0 RC. The primary objective of the phase III component is to confirm that TNT followed by W&W, which is less invasive and may avoid the need for colostomy, is non-inferior to the standard treatment of surgical resection for RC by comparing the 5-year overall survival (OS) rate in the TNT and W&W group with that in a historical control group.

**Fig. 1:**
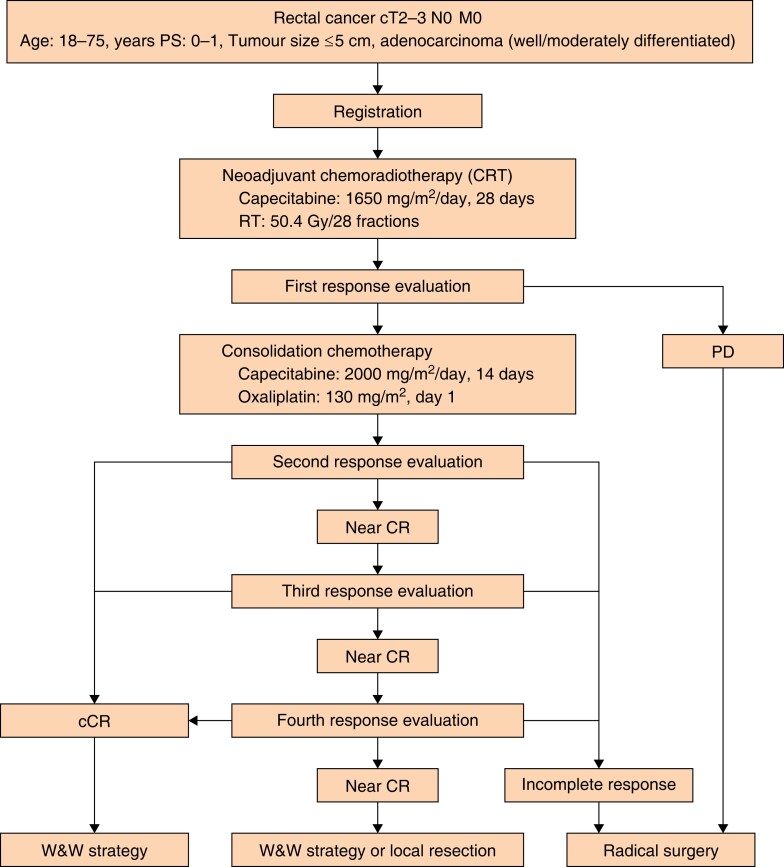
Schema for the JCOG2010 study Patients initially undergo neoadjuvant chemoradiotherapy (using capecitabine and a total irradiation of 50.4 Gy/28 fractions), and thereafter the first response evaluation is performed. If there is no apparent progressive disease or new tumour lesion, consolidation chemotherapy (four cycles of CAPOX (capecitabine/oxaliplatin)) will be implemented. After consolidation chemotherapy, three treatment strategies, including W&W, local resection, or radical surgery, will be considered according to the results of the response evaluation (that is cCR, near complete response, or incomplete response). W&W must be carried out until disease progression or relapse is detected. PS, performance status; RT, radiotherapy; PD, progressive disease; CR, complete response; W&W, watch-and-wait strategy.

A randomized phase III trial comparing the efficacy of TNT and W&W with that of surgical resection with lymph node dissection was not opted for, in view of the findings of JCOG0212, which reported a 5-year OS rate of 95.3 per cent for patients with cT3 N0 M0 disease and a tumour diameter of less than or equal to 5 cm^3,17,18^. It is unlikely that the 5-year OS rate would be less than 95 per cent in the standard treatment group, given that cT2 N0 M0 is associated with a better prognosis than cT3 N0 M0. Even without randomized controls, the efficacy of the study treatment could be established if the 5-year OS rate in the TNT and W&W group is non-inferior to 95 per cent. Therefore, a randomized trial would be of limited value, and a single-arm study design has been selected. The study schema is shown in *Fig. 1*.

In this study, the response will be evaluated according to the Memorial Sloan Kettering Cancer Center (MSKCC) criteria, which were initially proposed by the MSKCC and used in OPRA, a phase II trial that assessed the usefulness of W&W^19^. These criteria pertain only to primary tumours in the rectum and regional lymph nodes, so cannot be used to assess other lesions. Patients are classified into three response categories: cCR, near complete response (near CR), or incomplete response.

### Participants

#### Eligibility criteria

Patients must meet all of the following criteria: endoscopic biopsy from a primary rectal lesion histologically diagnosed as tubular adenocarcinoma (well or moderately differentiated) or endoscopic biopsy from a primary lesion in the anal canal histologically diagnosed as adenocarcinoma (rectal type) and further diagnosed as well or moderately differentiated; the tumour is located predominantly in the upper rectum, lower rectum, or anal canal; the lower margin of the tumour is located between the peritoneal reflection and the anal verge (lower rectum–anal canal); comprehensive diagnosis of cT2–cT3 by abdominal and pelvic contrast CT and pelvic contrast MRI with a slice thickness of less than or equal to 5 mm (Japanese Society for Cancer of the Colon and Rectum guidelines, ninth edition)—if contrast-enhanced CT or contrast-enhanced MRI is not possible because of allergy, renal dysfunction, or bronchial asthma, plain CT or plain MRI is acceptable; no lymph node satisfying either a or b below is present on comprehensive diagnosis by contrast-enhanced CT of the chest, abdomen, and pelvic region with a slice thickness of less than or equal to 5 mm and contrast-enhanced MRI of the pelvic region (a, 10 mm or more in short diameter; b, 7 mm or more in short diameter and one or more of the following: irregular margins, internal inhomogeneous low-signal areas shown on MRI, and circular (long to short diameter ratio less than 1.5)); no distant metastasis; maximum tumour diameter less than 5 cm on comprehensive diagnosis by contrast-enhanced CT of the abdomen and pelvis with a slice thickness of less than or equal to 5 mm, contrast-enhanced MRI of the pelvis, and endoscopy of the lower gastrointestinal tract; comprehensive diagnosis using colonoscopy and imaging studies (barium enema or abdominal/pelvic contrast CT or CT colonography) does not reveal multiple carcinomas (however, cTis and cT1a (cancer expected to remain in the submucosal layer and invade by less than 1000 µm) lesions that are expected to be curatively treated by endoscopic resection are not considered multiple carcinomas); aged between 18 and 75 years on the date of enrolment; Eastern Cooperative Oncology Group performance status 0 or 1; the patient does not wish to undergo surgical resection (that is the standard of care) as initial treatment, but wishes to undergo salvage treatment, including surgical resection, in the event of residual or recurrent disease; oral intake possible; no history of rectal resection (except for endoscopic resection), radiation therapy to the pelvic region, or any previous treatment, including for other types of cancer; the most recent laboratory test values within 14 days before registration include neutrophil count greater than or equal to 1500/mm^3^, haemoglobin greater than or equal to 9.0 g/dL, platelet count greater than or equal to 100 000/mm^3^, total bilirubin less than or equal to 2.0 mg/dL, aspartate transaminase less than or equal to 100 U/L, alanine transaminase less than or equal to 100 U/L, and serum creatinine less than or equal to 1.5 mg/dL; and able to provide written informed consent.

#### Exclusion criteria

Patients with any of the following are excluded: synchronous or metachronous (within 5 years) malignancy except for cancer with a 5-year relative survival rate of greater than or equal to 95 per cent, such as carcinoma *in situ*, intramucosal tumour, or early-stage cancer; infectious disease requiring systemic treatment; body temperature greater than or equal to 38.0°C at the time of registration; women who are pregnant, within 28 days postpartum, or lactating, and men whose partners wish to become pregnant; severe psychiatric disease affecting everyday life; receiving continuous systemic corticosteroids or immunosuppressive agents; poorly controlled diabetes mellitus; uncontrolled hypertension; history of unstable angina pectoris within 3 weeks or myocardial infarction within 6 months before registration; poorly controlled valvular heart disease, dilated cardiomyopathy, or hypertrophic cardiomyopathy; hepatitis B surface antigen positive; human immunodeficiency virus antibody positive; and having one or more of interstitial pneumonia, pulmonary fibrosis, or severe emphysema diagnosed by chest CT.

### Interventions

#### Neoadjuvant chemoradiotherapy

In this study, nCRT consists of oral capecitabine at a dose of 1650 mg/m^2^/day for 28 days and irradiation of the pelvic cavity at a dose of 45 Gy/25 fractions with boost irradiation of the primary tumour at 5.4 Gy/3 fractions. Intensity-modulated radiation therapy (IMRT) and three-dimensional conformal radiation therapy (3D-CRT) can be used in medical facilities that comply with the requirements for use of these modalities. It is permissible to apply IMRT at a dose of 45 Gy/25 fractions to the pelvic region followed by a switch to 3D-CRT at a dose of 5.4 Gy/3 fractions to intensify treatment of the primary tumour. It is not deemed acceptable to change the method of irradiation while irradiating the pelvic region.

#### Consolidation chemotherapy

After completion of nCRT, the first response evaluation is performed on days 8–21, with the final day of irradiation being day 0. If there is no apparent progressive disease or new tumour lesion, consolidation chemotherapy will be implemented. Conversely, if tumour progression is detected, radical surgery will be undertaken.

Consolidation chemotherapy involves administration of four cycles of CAPOX, which comprises capecitabine at a dose of 2000 mg/m^2^/day for 14 days in combination with oxaliplatin at a dose of 130 mg/m^2^ on day 1, starting on day 21 after the last irradiation. Even if consolidation chemotherapy is not possible because of nCRT-related adverse events, it may be initiated as a protocol therapy until day 35.

#### Evaluation of response after consolidation chemotherapy to determine whether the subsequent treatment strategy should be a watch-and-wait strategy, radical surgery, or local resection

After consolidation chemotherapy, the second response evaluation is performed on days 28–43, with day 0 being the last day of the fourth course of CAPOX. For patients who have not completed consolidation chemotherapy, the second response evaluation is performed on days 28–43, with the final day of consolidation chemotherapy being day 0.

If the second response evaluation indicates cCR, W&W is chosen. However, if the response is deemed to be incomplete, radical surgery is performed. Subsequent response evaluations are scheduled to be conducted no more than twice every 8 weeks for patients achieving near CR. According to the results of the response evaluation, one of the three types of treatment, namely W&W, local resection, or radical surgery, is selected. If distant metastasis occurs, the protocol treatment is terminated, and post-study treatment is not regulated.

#### Watch-and-wait strategy: non-operative follow-up

W&W is initiated for patients who achieve cCR at any of the second to fourth response evaluations or near CR at the fourth response evaluation (regarded as the final response evaluation) and express willingness to undergo W&W after consulting with a primary physician. W&W should be carried out until disease progression or relapse is detected. After the final response evaluation, follow-up should be conducted at 12-week intervals for 2 years and at 24-week intervals for 3 years thereafter. In the event of recurrence or progression of the primary tumour or involvement of regional lymph nodes, radical surgery is recommended as off-protocol treatment.

#### Radical surgery

For patients who are judged to have an incomplete response at any response evaluation or near CR at the final evaluation, but express willingness to undergo radical surgery after consulting with a primary physician, the procedure will be carried out within 2–14 weeks from the final evaluation provided that both of the following criteria are met: the patient should be suitable for R0 resection, as determined by preoperative imaging tests; and the following laboratory criteria are met within the 14 days preceding radical surgery—white blood cell count greater than or equal to 3000/mm^3^ and platelet count greater than or equal to 100 000/mm^3^.

If the first response evaluation reveals tumour progression or distant metastasis that is considered amenable to R0 curative surgery, radical surgery is recommended as off-protocol treatment. Off-protocol radical surgery is also recommended for patients in whom nCRT is discontinued for whatever reason, but curative R0 resection is still considered feasible.

#### Procedures

Radical surgery may consist of total mesorectal excision or tumour-specific mesorectal excision, both of which are considered standard procedures. Addition of lateral lymph node dissection is not specified. Open, laparoscopic, or robotic-assisted procedures are all acceptable.

#### Local resection

For patients who are judged to have near CR at the final response evaluation and are willing to undergo local resection after consulting with a primary physician, the procedure will be performed within 2–14 weeks from the date of the final response evaluation, provided that the following preoperative laboratory criteria are met: white blood cell count greater than or equal to 3000/mm^3^; and platelet count greater than or equal to 100 000/mm^3^.

#### Procedures

Local resection will be performed as transanal surgical resection using an appropriate technique (for example transanal endoscopic microsurgery or transanal minimally invasive surgery) or as transanal endoscopic resection (for example endoscopic mucosal resection or endoscopic submucosal dissection).

### Outcomes

#### Phase II component

The primary endpoint of the phase II component is the proportion of patients with cCR at the final response evaluation (as assessed by central review to enhance the robustness of the final assessment).

The secondary endpoints in phase II are the proportion of patients with a clinical/near CR at the final response evaluation (by central review), the proportion of patients who do not undergo resection of the rectum, adverse events, the incidence of surgical morbidities, and the concordance of response evaluation between the central review and the institutional in-house review.

The final response evaluation is the point when a patient achieves cCR or the fourth response evaluation, whichever is earliest. The proportions of patients with cCR or cCR/near CR are defined according to the MSKCC criteria and include all eligible patients. The proportion of patients who do not undergo rectal resection is defined as the proportion treated with W&W or local resection and includes all eligible patients. Adverse events associated with preoperative CRT and consolidation chemotherapy are assessed in accordance with the Common Terminology Criteria for Adverse Events (CTCAE) version 5.0. Surgical morbidities are evaluated according to CTCAE version 5.0 and the Clavien–Dindo classification^20^. Concordance of response evaluation between the central review and the institutional in-house review is calculated.

#### Phase III component

The primary endpoint of the phase III component is OS in all enrolled patients. OS is defined as the time interval between registration and death from any cause, and data are censored on the last day the patient is alive. The secondary endpoints include progression-free survival, local progression-free survival, rectal resection-free survival, colostomy-free survival, the proportion of patients with cCR at the final response evaluation (by central review), the proportion of patients with cCR/near CR at the final response evaluation (by central review), the proportion of patients who do not undergo rectal resection, adverse events, incidence of surgical morbidities, concordance of response evaluation between the central review and the institutional in-house review, the proportion of patients with R0 resection among those who undergo radical surgery, the proportion of patients with tumour progression among those who undergo W&W, defecation and anal function (Wexner and Low Anterior Resection Syndrome scores^21,22^), urinary outcomes (International Prostate Symptom score^23^), and sexual function in male patients (score on the five-item version of the International Index of Erectile Function^24^). Progression-free survival is defined as the time interval between registration and disease progression (including events after cCR/near CR or radical surgery) or death from any cause. The data are censored on the last day the patient is alive without any evidence of disease progression. Wexner and International Prostate Symptom scores and the score on the five-item version of the International Index of Erectile Function^24^ are evaluated before treatment and at 1 year and 5 years after registration.

### Participant timeline and follow-up schedule

Written informed consent will be obtained before registration from all patients who meet the inclusion criteria and do not fulfil any of the exclusion criteria. All study participants will be followed up for at least 5 years after enrolment. The primary endpoint will be analysed at 6 months after completion of patient enrolment in the phase II component and 5 years after completion of patient enrolment in the phase III component. Patients who are judged to have cCR or near CR at the final response evaluation and undergo W&W will be evaluated by chest CT, enhanced abdominal and pelvic CT and MRI, rectal examination, lower gastrointestinal endoscopy, and measurement of tumour markers (carcinoembryonic antigen and carbohydrate antigen 19-9) at 12-week intervals for 2 years after the final response evaluation and at 24-week intervals for 3 years thereafter. Patients who undergo radical surgery or local resection will be assessed by chest CT, enhanced abdominal and pelvic CT, and measurement of carcinoembryonic antigen and carbohydrate antigen 19-9 levels at 12-week intervals for 2 years after the final response evaluation and at 24-week intervals for 3 years thereafter. Colostomy status and anal function (preserved or non-preserved) will be evaluated at 6-month intervals for 3 years after initiation of irradiation and at 12-month intervals thereafter.

### Sample size and statistical methods

Mindful that the patients in this study have a higher probability of achieving cCR owing to the regulated eligibility criteria and that some patients who would have been deemed to have cCR in previous studies might be regarded as having near CR because of the more stringent assessment by central review in the JCOG2010 study, the threshold cCR rate was set at 20 per cent in the phase II component. The expected cCR rate was then set at 40 per cent based on consensus of members of the JCOG Colorectal Cancer Study Group. With a one-sided α value of 5 per cent and 80 per cent power, the required sample size was calculated to be 38 patients. Allowing for the fact that some patients may become ineligible, the planned sample size for the phase II component of the study was finally set at 40 patients.

In JCOG0212, the 5-year OS rate for patients with cT3 N0 M0 lower RC of less than or equal to 5 cm was 95.3 per cent. Although the present study includes patients with cT2 N0 M0 disease, which has a better prognosis than cT3 N0 M0, it may be challenging to significantly improve the prognosis given the inherently favourable prognosis of this population. Therefore, the expected 5-year OS rate was set at 95 per cent, and the threshold 5-year OS rate was set at 87 per cent considering that radical surgery would not be required in about half of the patients because of TNT and W&W. Thus, the final analysis will include the phase II population. Assuming a one-sided α value of 5 per cent and 80 per cent power, the sample size needed was calculated to be 98 patients. Considering that some patients may be lost to follow-up, the planned sample size is 105 patients (phase II, 40 patients; phase III, 65 patients). The expected accrual interval is 3.5 years, and the expected follow-up duration is 5 years after completion of enrolment. All statistical analyses will be performed at the JCOG Data Centre.

### Ethics and dissemination

The study protocol was approved by the JCOG Protocol Review Committee in April 2022 and by the National Cancer Centre Hospital Certified Review Board in June 2022. The trial is registered at the Japan Registry of Clinical Trials as jRCTs031220288 (https://jrct.niph.go.jp/en-latest-detail/jRCTs031220288). All patients will receive information to assist with decision-making regarding whether to participate in the study. Consent to publication is included in the general consent form, and each participant’s data will be handled anonymously. All participant information will be stored at the JCOG Data Centre.

## Discussion

This article describes the context, methodology, and rationale of the multi-institutional, single-arm phase II/III JCOG2010 trial, which focuses on patients with cT2–3 N0 M0 RC and a tumour diameter of less than or equal to 5 cm who are likely to achieve cCR after TNT.

In this study population, radical surgery involves resection of the rectum, which results in significant deterioration of postoperative QoL in almost all patients. In JCOG0212, early dysuria was observed in 58 per cent of patients who underwent total mesorectal excision, and erectile and ejaculatory dysfunction was frequently reported in men^3^. A preoperative survey of patients with colorectal cancer found that 76 per cent hoped that surgery would be curative, 78 per cent were unwilling to receive a permanent stoma, and 74 per cent wished to avoid postoperative complications^25^. Therefore, postoperative QoL should be considered one of the essential endpoints in this population. However, potential risks associated with the TNT strategy include the increased likelihood of local recurrence, possible tumour progression requiring extended surgery, and an elevated risk of adverse events when performing definitive surgery after TNT. Therefore, it is crucial to consider the balance between efficacy and adverse events when interpreting the results of the JCOG2010 study^26^.

Regarding the interval between completion of TNT and response evaluation, the pCR rate in the Lyon R90-01 trial was higher in the group with a 6–8-week interval between preoperative irradiation and surgery than in the group with a 2-week interval (26 *versus* 10.3 per cent respectively)^27^. In the GRECCAR 6 trial, however, there was no significant difference in the pCR rate according to whether nCRT was followed by surgery at 7 or 11 weeks^28^. Furthermore, previous studies of the efficacy of rectum-preserving therapy have typically performed response evaluations at 12–38 weeks after initiation of treatment, whereas studies that have used the cCR rate as the endpoint have generally performed response evaluations at 24 weeks after initiation of treatment^12^. In the present study, the second response evaluation, which offers the first opportunity to assess cCR, near CR, or incomplete response according to the MSKCC criteria, will be performed 24–26 weeks after initiation of treatment. The third and fourth response evaluations will be performed 32 and 40 weeks after initiation of treatment. Therefore, it can be assumed that the effect of TNT will be adequately observed.

The cCR rate achieved by nCRT for cT3N0 rectal carcinoma, which is associated with feasibility of preserving the rectum, was noted to be 54.2 per cent when the two-classification system (cCR or incomplete response) was used^29^. Despite focusing on patients with cT2 N0 M0 disease, the present study considers addition of consolidation chemotherapy as TNT, which could potentially augment the cCR rate. However, the assessment criteria for treatment response entail a three-classification system (cCR, near CR, or incomplete response). It is plausible that some patients in this study who would be previously identified as having cCR could be classified as having near CR. The expected cCR and near CR rates in this study have therefore been set at 40 and 20 per cent respectively. In view of the lower probability that patients categorized as having near CR would opt for radical surgery and data from a previous report^30^, around 60 per cent of patients classified as having near CR would be expected to select W&W and 40 per cent would opt for local resection. In summary, W&W would be performed for 52 per cent of patients and local resection for 8 per cent of patients in the JCOG2010 study. Assuming that approximately 40 per cent of local resection cases and 20 per cent of W&W cases will undergo radical surgery for residual tumour or local progression, rectal preservation would ultimately be expected in 45.4 per cent of patients in the present study.

This study will include a longitudinal evaluation of anal and defecation function, urinary function, and sexual function in men. If rectal preservation by TNT and W&W is practicable, these functions may be expected to be preserved. However, there is currently no information on the degree to which TNT affects these functions, and the longitudinal analysis may provide fundamental data for clinical research and enable informed decision-making when TNT is implemented in clinical practice. An ancillary study is also planned to assess the potential value of circulating tumour DNA as a marker for therapeutic and prognostic purposes in patients with RC who are treated with TNT followed by W&W. Correlating the findings of the ancillary study with those of the present study could significantly influence clinical practice and advance the field of personalized and precision medicine.
